# Saccadic Palsy following Cardiac Surgery: Possible Role of Perineuronal Nets

**DOI:** 10.1371/journal.pone.0132075

**Published:** 2015-07-02

**Authors:** Scott D. Z. Eggers, Anja K. E. Horn, Sigrun Roeber, Wolfgang Härtig, Govind Nair, Daniel S. Reich, R. John Leigh

**Affiliations:** 1 Department of Neurology, Mayo Clinic, Rochester, Minnesota, United States of America; 2 Institute of Anatomy and Cell Biology I, Ludwig-Maximilians University, Munich, Germany; 3 German Center for Vertigo and Balance Disorders, Ludwig-Maximilians University, Munich, Germany; 4 Institute for Neuropathology and Prion Research, Ludwig-Maximilians University, Munich, Germany; 5 Paul Flechsig Institute for Brain Research, University of Leipzig, Leipzig, Germany; 6 National Institute of Neurological Disorders and Stroke, National Institutes of Health, Bethesda, Maryland, United States of America; 7 Department of Neurology, Case Western Reserve University, Cleveland, Ohio, United States of America; State University of New York Downstate Medical Center, UNITED STATES

## Abstract

**Objective:**

Perineuronal nets (PN) form a specialized extracellular matrix around certain highly active neurons within the central nervous system and may help to stabilize synaptic contacts, promote local ion homeostasis, or play a protective role. Within the ocular motor system, excitatory burst neurons and omnipause neurons are highly active cells that generate rapid eye movements – saccades; both groups of neurons contain the calcium-binding protein parvalbumin and are ensheathed by PN. Experimental lesions of excitatory burst neurons and omnipause neurons cause slowing or complete loss of saccades. Selective palsy of saccades in humans is reported following cardiac surgery, but such cases have shown normal brainstem neuroimaging, with only one clinicopathological study that demonstrated paramedian pontine infarction. Our objective was to test the hypothesis that lesions of PN surrounding these brainstem saccade-related neurons may cause saccadic palsy.

**Methods:**

Together with four controls we studied the brain of a patient who had developed a permanent selective saccadic palsy following cardiac surgery and died several years later. Sections of formalin-fixed paraffin-embedded brainstem blocks were applied to double-immunoperoxidase staining of parvalbumin and three different components of PN. Triple immunofluorescence labeling for all PN components served as internal controls. Combined immunostaining of parvalbumin and synaptophysin revealed the presence of synapses.

**Results:**

Excitatory burst neurons and omnipause neurons were preserved and still received synaptic input, but their surrounding PN showed severe loss or fragmentation.

**Interpretation:**

Our findings support current models and experimental studies of the brainstem saccade-generating neurons and indicate that damage to PN may permanently impair the function of these neurons that the PN ensheathe. How a postulated hypoxic mechanism could selectively damage the PN remains unclear. We propose that the well-studied saccadic eye movement system provides an accessible model to evaluate the role of PN in health and disease.

## Introduction

Saccades are rapid conjugate eye movements that bring target images onto the fovea for exploring visual scenes, reading, and resetting the eyes during optokinetic or vestibular nystagmus. Two critical components of the brainstem saccade network are premotor burst neurons (PBN) and omnipause neurons (OPN). PBN include excitatory burst neurons (EBN) and inhibitory burst neurons (IBN) firing up to 1,000 spikes/s during saccades [1]; at other times they are silent. PBN in the paramedian pontine reticular formation (PPRF) generate horizontal saccades [2] and in the rostral interstitial nucleus of the medial longitudinal fasciculus (RIMLF) vertical saccades [3]. In contrast, OPN in the nucleus raphe interpositus (RIP) [4] inhibit both PBN populations by firing continuously except during saccades [5,6]. The pathways for pursuit, vestibular, and vergence eye movements are distinct from those of the saccadic system in that they do not depend critically upon PBN and OPN.

Perineuronal nets (PN) are condensed, extracellular matrices of macromolecules ensheathing fast-firing neurons such as PBN and OPN. PN consist of a hyaluronic acid backbone attached to glycoproteins and chondroitin sulfate proteoglycans (CSPG) such as aggrecan (ACAN) and hyaluronan and proteoglycan link protein (HPLN1) [7]. PN may form a specialized microenvironment to stabilize synaptic contacts, promote neuroplasticity and neuroprotection, or serve as rapid potassium ion exchangers [8]. PN frequently enwrap fast-firing GABAergic neurons expressing parvalbumin [9]. PBN and OPN stand out within the brainstem reticular formation by being uniquely ensheathed by PN [10].

Infarction, metabolic or degenerative disorders targeting PBN or OPN may slow or abolish saccades [11–13]. Drawing on basic studies [14], neuromimetic models for brainstem saccade generation have been developed and successfully applied to understand saccadic abnormalities [15]. However, one disorder stands apart as an abiding mystery: the syndrome of saccadic palsy following cardiac or aortic surgery. Hanson et al [16] described a 31-year-old man undergoing aortic aneurysm and valve repair complicated by bleeding and hypotension who awoke with slow saccades horizontally and vertically and absent nystagmus quick phases. The visual system, pursuit, vergence, and vestibulo-ocular reflex were normal. Two months postoperatively he died of septicemia. Autopsy revealed focal neuronal necrosis, axonal loss and astrocytosis in the PPRF region. Ocular motor nuclei, medial longitudinal fasciculi, midbrain, and frontal eye fields appeared normal.

Similar cases have been reported without neuropathologic evaluation [17–26], typically with aortic valve or root surgery requiring cardiopulmonary bypass and hypothermic circulatory arrest. MRIs have not shown brainstem infarcts. Thus, the process causing selective saccadic palsy following cardiac surgery remains uncertain. Although ischemic damage to PBN and OPN remains possible, we recently studied one such patient in whom autopsy demonstrated preserved PBN and OPN [27]. Since experimental work has demonstrated that PN are vulnerable to ischemia [28], we investigated whether damage to brainstem saccadic network PN could account for her selective saccadic palsy.

## Patients and Methods

A previously reported [29] healthy 50-year-old woman underwent otherwise uncomplicated aortic valve replacement for an incidentally-discovered ascending aortic aneurysm. Upon awakening from anesthesia she was unable to redirect her gaze and began using head movements to facilitate gaze shifts. She had no dysarthria, dysphagia, or gait instability. She was discharged and had no problems except her visual complaints for 3 months when she developed complex partial seizures that responded to levetiracetam.

Ten months postoperatively, general neurological examination was notable only for diffuse hyporeflexia. Visual acuity, pupils, visual fields, and fundoscopic examination were normal. Straight-ahead fixation was steady, and no saccadic intrusions or nystagmus were seen with ophthalmoscopy. She made no reflexive saccades, and volitional saccades consisted of extremely slow eye movements that eventually reached the target, except for slightly faster downward saccades (S1 Movie). Pursuit was smooth and full in range both horizontally and vertically, even at higher frequencies. Vergence was normal. When she viewed a horizontally rotating optokinetic drum, her eyes became fixed laterally in the orbits without any corrective quick phases. With vertical optokinetic stimulation, she made a few downbeats of nystagmus with upward optokinetic drum, but made no upward quick phases. Visually enhanced vestibulo-ocular reflex and head impulse testing were normal. Torsional head rolling produced normal ocular counter-rolling but without any torsional quick phases. Fixation suppression of the vestibulo-ocular reflex during smooth eye-head tracking was intact. To make rapid head-free gaze shifts, she used exaggerated head turns associated with blinks; contraversive vestibular slow phases moved the eyes into the corner of the orbits until the head was maximally rotated, and then the eyes slowly moved toward the target.

Eye movements were recorded with video-oculography. Horizontal saccades were absent during a random saccade paradigm, but sinusoidal smooth pursuit was normal across all frequencies tested. Vertical eye movements demonstrated similar findings. Additionally, rotary chair sinusoidal vestibular and optokinetic testing generated normal vestibular slow phases and optokinetic ocular following reflex, but absent vestibular and optokinetic quick phases of nystagmus.

The patient was last examined two years later and was clinically unchanged except for some emerging memory complaints. She remained on warfarin for her mechanical valve. Subsequently, she developed cirrhosis attributed to increasing alcohol abuse. Memory loss remained mild. Eight years after her cardiac surgery, with an increased bleeding diathesis from over-anticoagulation and liver dysfunction, she developed massive hematemesis and melena from a bleeding peptic ulcer, profound anemia, shock, renal failure, and somnolence. She expired in hospice one week later.

**Fig 1:**
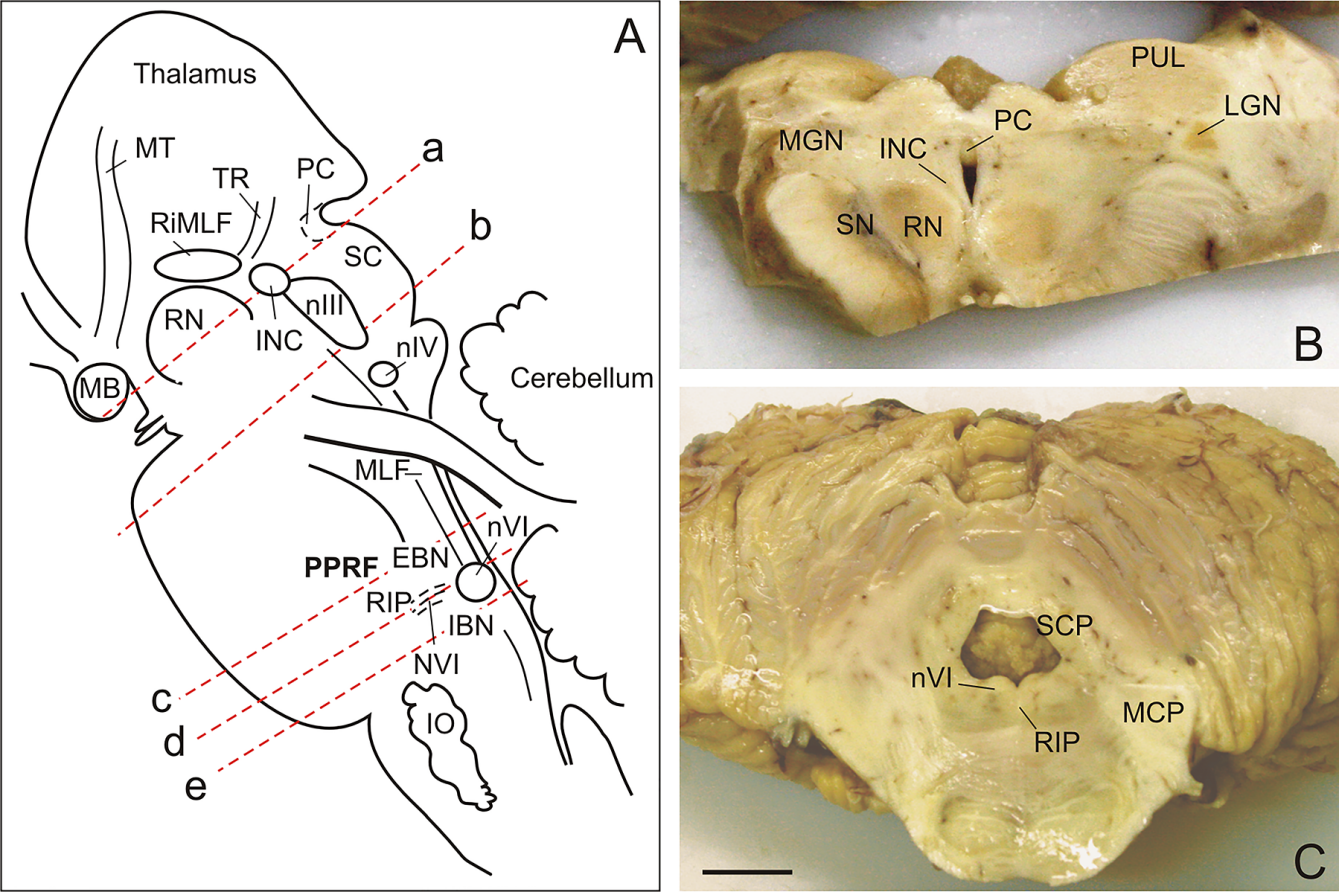
Brainstem cutting planes and transverse sections. (A) Brainstem sagittal view demonstrating cutting planes. The blocks containing the rostral interstitial nucleus of the medial longitudinal fascicle (RIMLF), the oculomotor nucleus (nIII), the paramedian pontine reticular formation (PPRF) including the excitatory (EBN) and inhibitory burst neurons (IBN), the nucleus raphe interpositus (RIP) containing omnipause neurons (OPN), and the abducens nucleus (nVI), were cut in series of 10μm and 5μm thick sections. (B) Caudal view of the block containing the RIMLF. (C) Caudal view of the block containing the PPRF and OPN region. Scale bar B,C = 1cm. INC, interstitial nucleus of Cajal; IO, inferior olive; MB, mammillary body; LGN, lateral geniculate nucleus; MCP, medial cerebellar peduncle; MGN, medial geniculate nucleus; MT, mammillothalamic tract; nIV, trochlear nucleus; NVI, abducens nerve; PC, posterior commissure; RN, red nucleus; TR, tractus retroflexus; PC, posterior commissure; PUL, pulvinar; SC, superior colliculus; SCP, superior cerebellar peduncle; SN, substantia nigra.

The whole brain was collected the day of death and fixed in 10% formalin for 2 weeks. Next, the brain was suspended in Fomblin (Solvay Solexis, West Deptford, NJ) for *ex vivo* 7 tesla MRI, as previously described [27]. After refixation in formalin, the brain was dissected, and the brainstem was cut transversely into several 2cm thick slices, which were embedded in paraffin. The blocks containing the RIMLF, the oculomotor nucleus, the PPRF including EBN and OPN, the abducens nucleus, and the IBN were cut in series of 10μm and 5μm thick sections (Fig 1A–1C). For neuropathological analysis, sections of each area were deparaffinized and stained with hematoxylin & eosin, cresyl violet and Luxol fast blue periodic acid-Schiff (LFB-PAS; myelin staining) using standard techniques. Immunocytochemical staining with polyclonal rabbit anti- glial fibrillary acid protein (GFAP, 1:3000, Dakocytomation; Glostrup, Denmark; N1506, RRID:AB_10013482) and mouse monoclonal anti-human HLA-DP, DQ, DR-antigen (clone CR3/43 1:100, Dakocytomation; Glostrup, Denmark; F081701, RRID: AB_578680) was performed to reveal reactive gliosis and activated microglia, respectively.

After deparaffinizing and antigen retrieval by boiling the sections 3x10 minutes in a microwave in 0.01M citrate buffer (pH 6) the slides were transferred to 0.1M phosphate-buffered saline (PBS; pH 7.3). Saccade-related neurons were identified by immunostaining with mouse monoclonal antibodies directed against non-phosphorylated neurofilaments (NP-NF, Covance; Emeryville, USA; SMI-32P; clone SMI32; RRID:AB_2314904; 1:5000) and the calcium-binding protein parvalbumin (PAV; Swant, Marly, Switzerland; 235, RRID:AB_10000343, 1:2500). Antigen binding sites were detected with incubation with biotinylated horse antibodies recognizing mouse IgG (Vector Laboratories; Burlingame, USA; BA2000, RRID:AB_2313581; 1:200) for 2 h at room temperature. Following 3 buffer washes and an 1 h incubation with ExtrAvidin-peroxidase conjugates (EAP; Sigma; Taufkirchen, Germany; E-2886; 1:1000) immunoreactivities were visualized with nickel-enhanced diaminobenzidine (DAB-Ni) and H_2_O_2_ resulting in a bluish-black reaction product [30].

PN were detected by immunostaining for different components with either mouse monoclonal anti-cat chondroitin sulfate proteoglycan (CSPG; clone 301; Millipore; MAB5284, RRID:AB_2219944; 1:500), or anti- human aggrecan core protein (ACAN; clone HAG7D4; Acris; Herford, Germany; SM1353P, RRID:AB_972582; 1:75), or polyclonal goat anti-human link protein (HPLN1 = CRTL1; R&D Systems; Wiesbaden, Germany; AF2608, RRID:AB_2116135; 1:400). Synapses were identified with affinity-purified rabbit antibodies directed against the presynaptic vesicle protein synaptophysin (Millipore, Billerica, USA; AB9272, RRID:AB_570874; 1:2000), which was visualized by the avidin-biotin-technique and DAB-Ni as described above. In selected sections, double immunoperoxidase staining was applied to concomitantly detect PN or synapses and PAV-positive saccade-related neurons. Thereby, PN-related antibodies or synaptophysin were revealed by immunoperoxidase double staining with bluish-black DAB-Ni followed by immunodetection of PAV with plain DAB producing a brown reaction product.

In selected sections, main components of PN were visualized by triple immunofluorescence labeling. The tissue was primarily blocked with 5% normal donkey serum in TBS (containing 0.3% Triton X-100) for 1 hour and then incubated with a cocktail consisting of polyclonal rabbit anti-CSPG (Chemicon, Temecula, USA; AB1918; 1:500) [10], polyclonal goat anti-HPLN1 (R&D Systems; Wiesbaden, Germany; AF2608, RRID:AB_2116135; 1:100) and mouse anti-ACAN (clone HAG7D4; Acris; Herford, Germany; SM1353P, RRID:AB_972582; 1:40) overnight. After washing, the sections were reacted for one hour with a mixture of highly purified Cy2-conjugated donkey anti-rabbit IgG (711-225-152, RRID:AB_2340612; Jackson ImmunoResearch, West Grove, USA), Cy3-conjugated donkey anti-goat IgG (705-165-003, RRID:AB_2340411 Jackson ImmunoResearch, West Grove, USA) and Alexa 647 conjugated donkey anti-mouse IgG (715-606-151, RRID:AB_2336929; Jackson ImmunoResearch, West Grove, USA) each at a concentration of 20 μg/ml in TBS containing 2% bovine serum albumin. Next, autofluorescence of the tissue was quenched by Sudan Black B treatment, and the sections were coverslipped with glycerol/gelatin (Sigma). A confocal laser scanning microscope using a 20 and 63 x oil objective (Leica TCS SP2, Heidelberg, Germany) was used for imaging.

For normal controls, the brains of 4 subjects from the Neurobiobank Munich who died without any history of ocular motor or relevant neurological disorders were evaluated. All cases underwent identical neuropathological examination, immunostaining with monoclonal mouse antibodies against human-PHF-Tau (clone AT8, Thermo Pierce Biotechnology, Rockford, USA, MN1020, RRID:AB_223647, 1:200), beta-Amyloid 17–24 (clone 4G8, Covance, Emeryville, USA, SIG-39220, 1:2000), alpha-synuclein (clone 42, BD Biosciences, Heidelberg, Germany, 610787, RRID:AB_398108, 1:10) and polyconal rabbit anti-TDP-43 (Proteintech Group, Chicago, USA, 10782-2-AP, RRID:AB_615042), as well as triple immunofluorescence labeling.

Control 1 (shown in figures) was a 62-year-old male who died of pancreatic cancer without brain metastases or hepatic encephalopathy whose neuropathological examination demonstrated small old hemorrhages in the adenohypophysis, arteriosclerosis, and Braak stage I of Alzheimer changes [31]. Control 2 was a 75-year-old female who died of heart failure after pneumonia whose neuropathological examination demonstrated a small infarct in the occipital white matter, arteriosclerosis, and stage I Alzheimer changes. Control 3 was a 71-year-old male who died of heart failure after pneumonia whose neuropathological examination demonstrated considerable atherosclerosis with mild stenosis of brainstem vessels and mild frontal and temporal lobe atrophy. Control 4 was a 67-year-old male with rectal cancer who died of heart failure whose neuropathological examination showed old infarcts in right occipital and frontal lobe.

Written consent was obtained from all patient and control subjects or the next of kin for use of these tissue samples in research. The patient provided written consent for video recording (that authorizes publication among other uses). The next of kin of the patient has provided written informed consent for brain autopsy and (as outlined in the PLOS consent form) to publish these case details and the video. Control samples were collected as part of the Neurobiobank Munich. The Neurobiobank Munich and this research project have been approved by the ethics committee of the Ludwig Maximilians University.

## Results

As previously reported, gross inspection of the brain was unremarkable (Fig 1B and 1C) and was consistent with *in vivo* and *ex vivo* 7 tesla MRI studies [27]. Atherosclerotic plaques were noted at the bifurcation of the basilar into the posterior cerebral arteries. Histologic examination revealed neuritic plaques and neurofibrillary tangles with tau (AT8) and β-amyloid (Aβ) staining consistent with stage IV of Braak’s staging of Alzheimer’s disease [31]. The tissue was immunonegative for TDP-43 and α–synuclein. The cerebellar cortex showed focal Purkinje cell loss and proliferation of Bergmann glia. A chronic right frontal lacunar infarct was noted. The right superior colliculus showed a cystic space with an accumulation of microglial cells, but without signs of prior hemorrhage or infarct. Chronic demyelination was seen in the dorsal columns of the cervical spinal cord.

**Fig 2:**
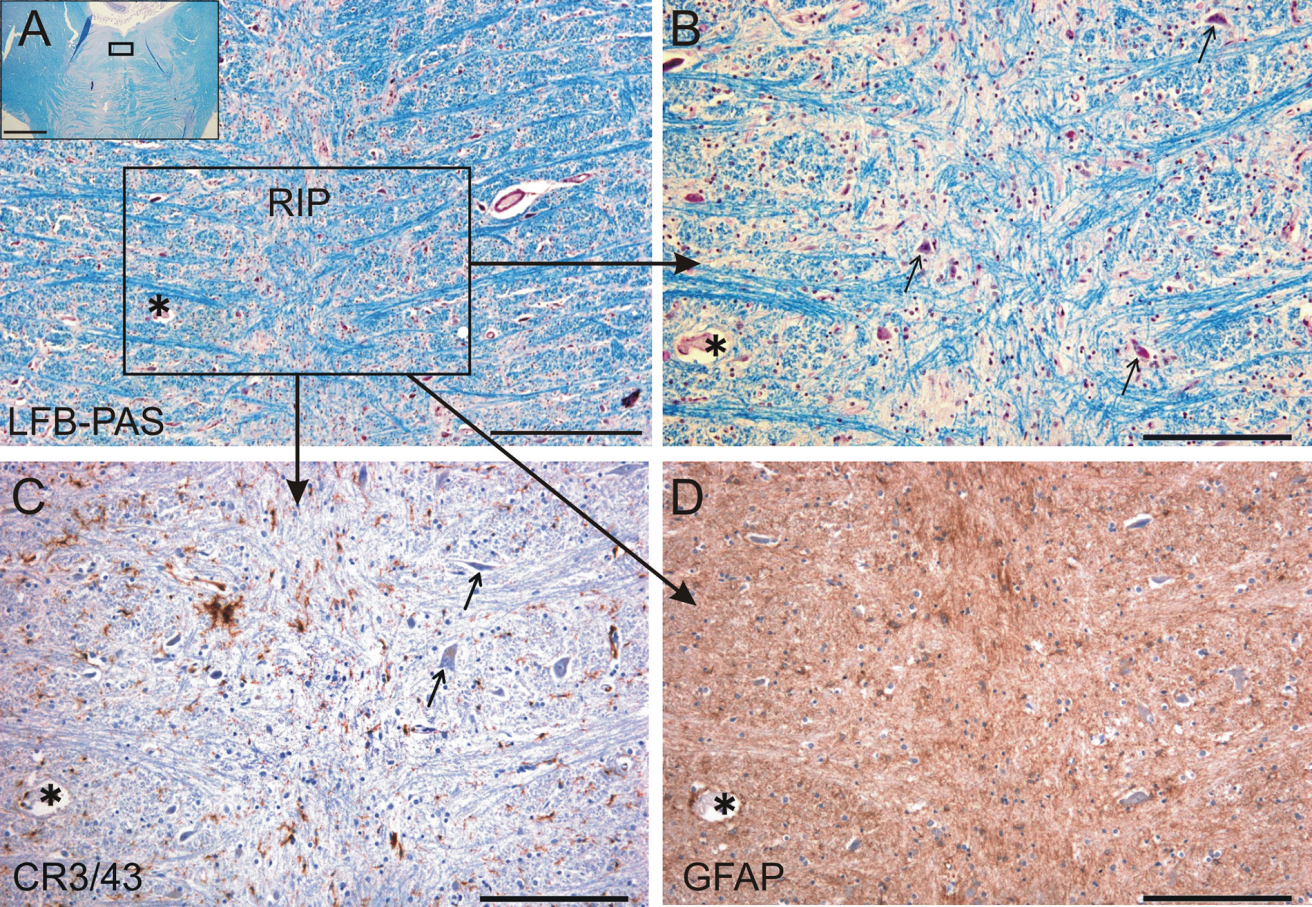
Histologically normal omnipause neurons. Omnipause neurons in the nucleus raphe interpositus (RIP) appear histologically normal (arrows). (A) Photograph of a transverse section of the pons at the level of RIP. The inset indicates the area shown in A. (B) LFB-PAS staining showed no evidence of demyelination. (C) CR 3/43 staining showed no abnormal microglial activation. (D) GFAP staining showed no reactive gliosis. The asterisk labels the same blood vessel in neighboring sections for orientation. LFB-PAS, Luxol fast blue periodic acid-Schiff; GFAP, glial fibrillary acidic protein. Scale bar A = 500μm, inset = 5mm; B-D = 200μm.

The gross and histologic examination of the pons in the region of the abducens and omnipause neurons found no lesion. Staining the OPN area with LFB-PAS, GFAP, and CR3/43 showed no evidence of demyelination, reactive gliosis, or abnormal microglia activation (Fig 2A–2D). Immunolabeling of markers in the RIMLF also revealed no abnormalities.

**Fig 3:**
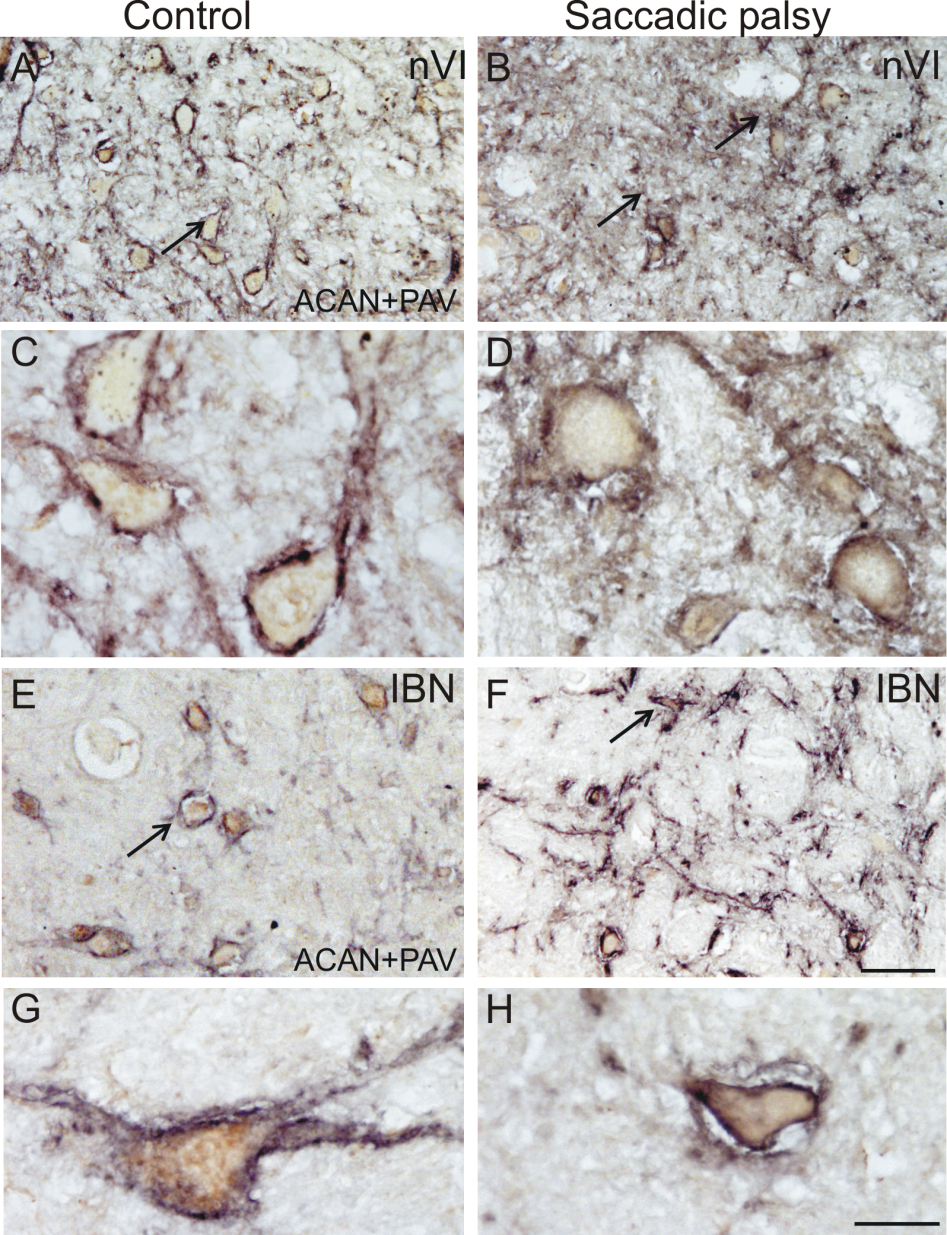
Normal abducens nucleus perineuronal net immunostaining. Detection of perineuronal nets by aggrecan (ACAN) immunolabeling in the abducens nucleus (nVI) appeared similar in control subject and patient specimens. Low power (A and B) and high power (C and D) views of parvalbumin-positive (PAV) abducens neurons (brown) ensheathed by aggrecan (ACAN)-immunoreactive perineuronal nets (black, arrows). In the tissue from the control (A and C) and from the patient (B and D), ACAN-stained perineuronal nets (arrows) appeared in unaltered manner. Low power (E and F) and high power (G and H) photographs of the inhibitory burst neurons (IBN) of the control (E and G) and patient (F and H) displaying similar perineuronal nets around PAV-positive IBN. Scale bars A,B,E,F = 100μm; C,D,G,H = 30μm.

Immunostaining patterns of HPLN1- and ACAN-based PN in the abducens nuclei (Fig 3A–3D) and around the PAV-positive inhibitory burst neurons (IBN) in the caudal PPRF appeared normal (Fig 3E–3H). Likewise, PN of cerebellar fastigial nucleus neurons were not altered compared to controls.

**Fig 4:**
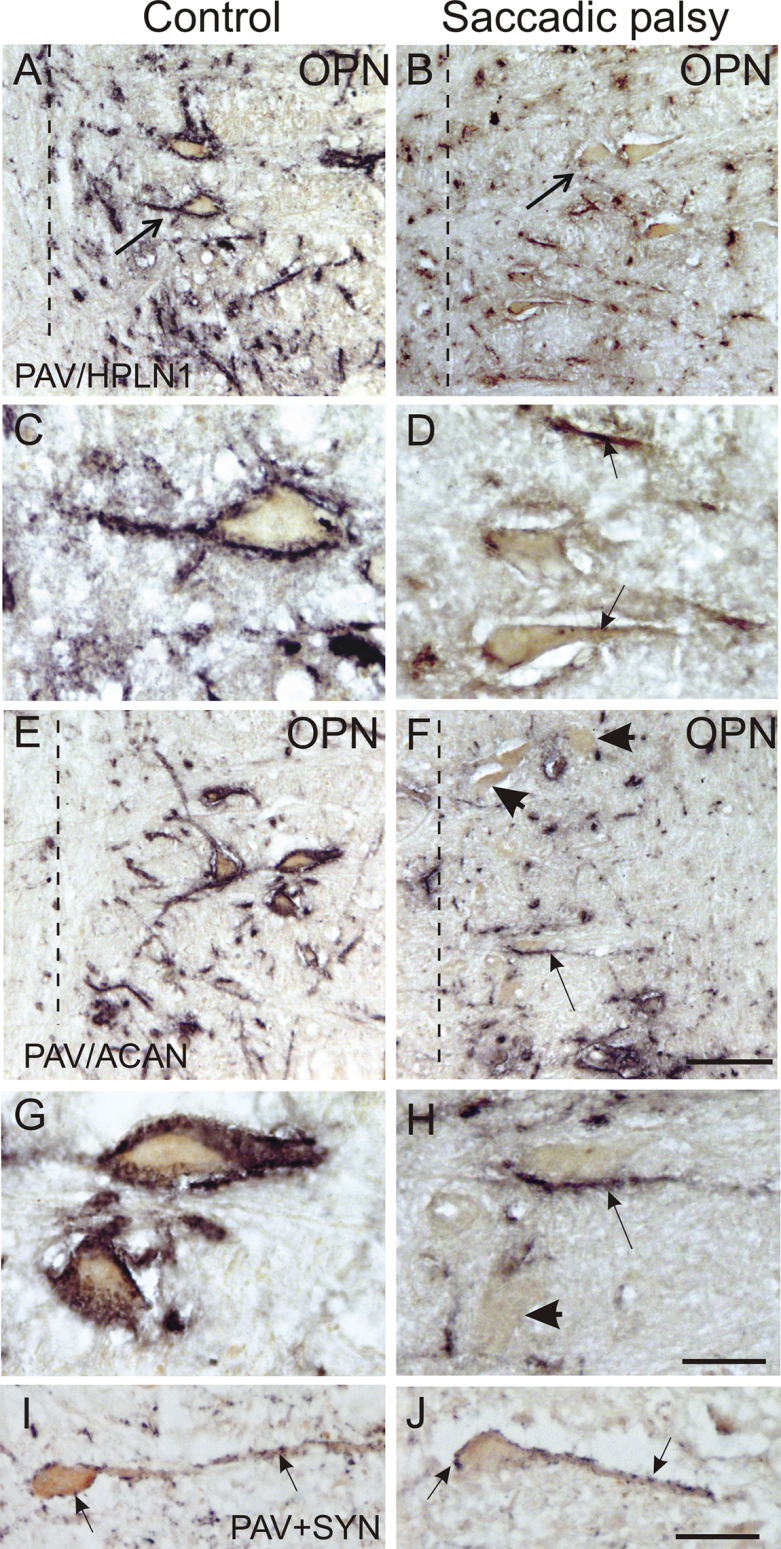
Absent or fragmented omnipause neuron perineuronal net immunostaining. Perineuronal nets around omnipause neurons (OPN) were absent or abnormal by all immunostaining methods, compared to a normal control subject. The left column demonstrates a control subject, whose parvalbumin (PAV)-positive OPN appear normal. Low- and high-power views of the control show normal-appearing perineuronal nets staining with HPLN1 (A, C, arrows) and aggrecan (ACAN) antibodies (E, G). The right column demonstrates OPN stained for PAV (B, D, F, H, arrows). Their perineuronal nets are either absent or fragmented when stained for HPLN1 (B, D, arrows) or ACAN (F, H, arrows). High-power views of PAV-positive OPN (brown) contacted by synaptophysin-positive terminals (black) in the control (I) and patient (J) appeared similar. Scale bars A,B,E,F = 100μm; C,D,G,H,I,J = 30μm. Dashed line (A, B, E, F) designates the midline.

**Fig 5:**
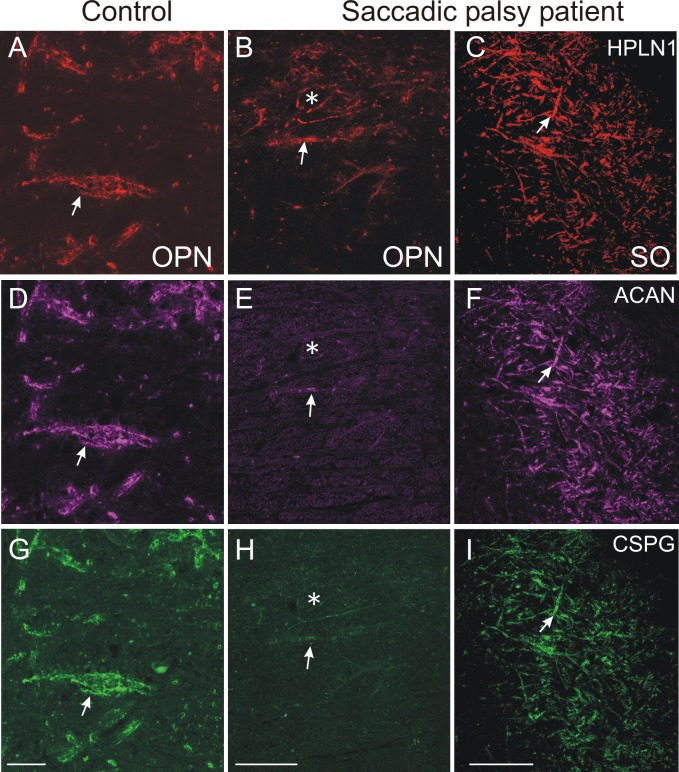
Absent or fragmented omnipause neuron perineuronal net triple immunofluorescence staining. Triple immunofluorescence staining for different components of perineuronal nets, revealed by a confocal laser scanning microscope. In the control case, omnipause neurons (OPN) are ensheathed by prominent perineuronal nets showing the same appearance with antibodies against the link protein (HPLN1), chondroitin sulfate proteoglycan (CSPG) and aggrecan (ACAN) (A, D, G, arrow). In the saccadic palsy patient, the neurons of the superior olive (SO) from the same sections as OPN are ensheathed by prominent perineuronal nets revealed by immunostaining of HPLN1, CSPG, and ACAN (C, F, I, arrows). However, around OPN (asterisk) in the patient, only HPLN1-based perineuronal nets can be detected, which appear fragmented (B, arrow). CSPG- and ACAN-immunostaining does not reveal perineuronal nets, but only few fragments along a few dendrites (E, H, arrow). Scale bars A,D,G = 20μm; B,C,E,F,H,I = 200μm.

In the nucleus raphe interpositus (RIP) of control subjects, all PAV-positive OPN were ensheathed by PN immunostained for ACAN, CSPG, or HPLN1 (Fig 4A, 4C, 4E and 4G). In the patient, the OPN could still be identified by their PAV-immunolabeling, but ACAN- or HPLN1-based PN around most putative OPN either were absent or appeared fragmented (Fig 4B, 4D, 4F and 4H). CSPG-immunoreactivity was barely visible or associated with diffuse cellular staining, indicating a dislocation of the proteins (not shown). As in the control case, the OPN of the patient were associated with synaptophysin-positive boutons, indicating that the neurons still received synaptic input (Fig 4I and 4J). To rule out misinterpretation of the PN appearance due to possible weak staining from methodological reasons, triple immunofluorescence labeling for the simultaneous detection of all three PN components in a single section was performed. As with single immunoperoxidase staining of the relevant markers, OPN in the control case were ensheathed by prominent PN stained with antibodies recognizing HPLN1, CSPG, and ACAN (Fig 5A, 5D and 5G). However, in the patient, only fragmented HPLN1-immunoreactive PN could be detected around OPN. ACAN and CSPG immunostaining failed to demonstrate any intact PN except for a few fragments along dendrites (Fig 5B, 5E and 5H). The strong immunolabeling of all PN components around neurons of the superior olive in the same section of the patient served as an internal control (Fig 5C, 5F and 5I).

**Fig 6:**
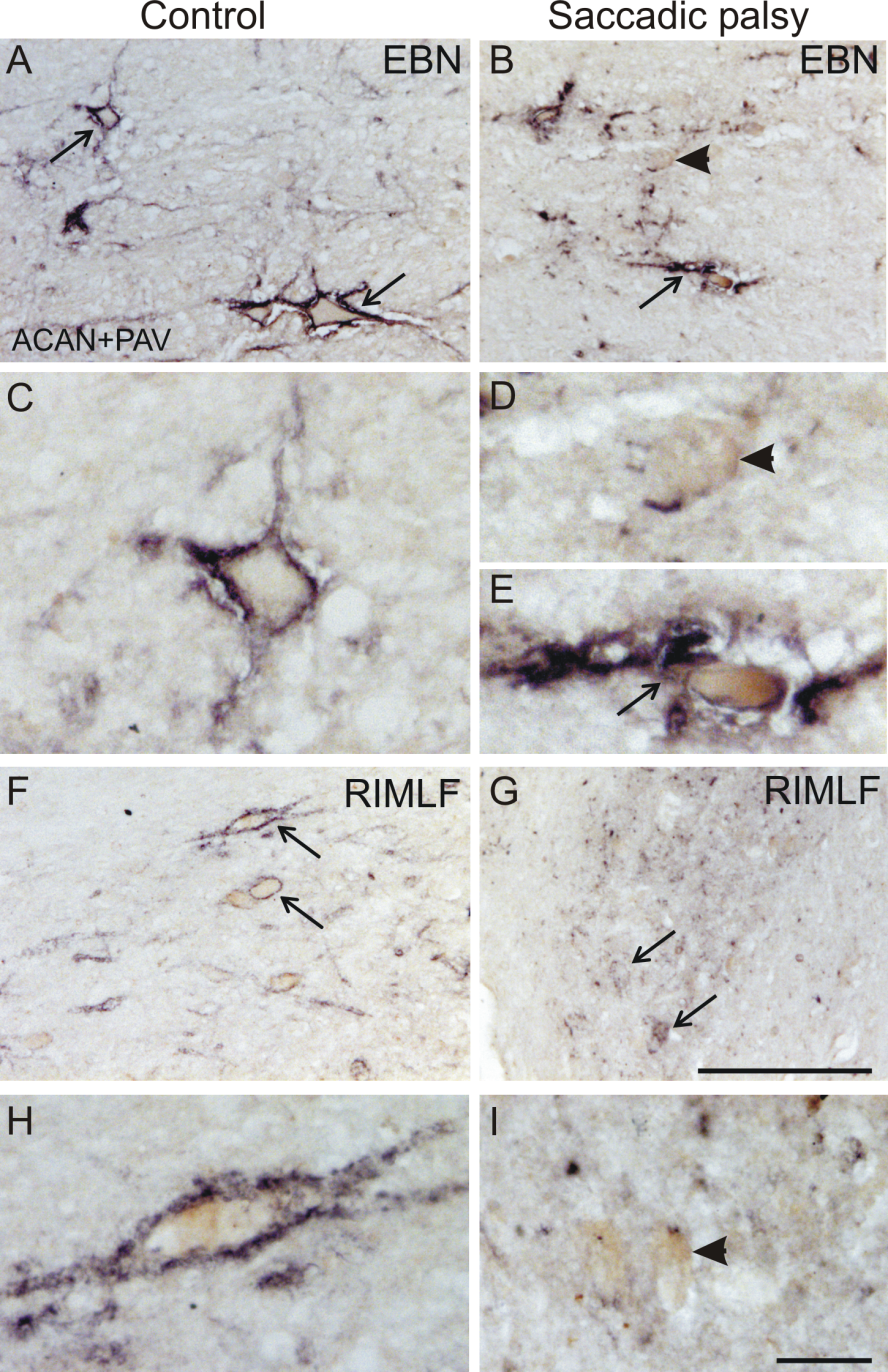
Fragmented burst neuron perineuronal net immunostaining. Parvalbumin-positive (PAV) putative burst neurons in the rostral interstitial nucleus of the medial longitudinal fasciculus (RIMLF) and in the paramedian pontine reticular formation (PPRF) were present in both the control and patient. Perineuronal nets appeared normal around burst neurons (EBN) in the PPRF (A, C, arrows) and RIMLF (F, H, arrows) in the control, here immunostained for aggrecan (ACAN). However, the patient’s burst neurons in the PPRF were ensheathed only by fragments of ACAN-based perineuronal nets (B, D, short arrow). Few PAV-positive neurons were found with preserved perineuronal nets (B, E, long arrow). Similarly, the PAV-positive neurons (brown) in the RIMLF of the control are enwrapped by distinct perineuronal nets (F, H, arrows), whereas only fragments are found in the RIMLF of the patient (G, short arrows). Most PAV-positive neurons are attached to few ACAN-positive fragments (I, short arrow). Scale bars: A,B,F,G = 200μm; C,D,E,H,I = 30μm.

The PAV-positive putative excitatory saccadic burst neurons in the PPRF and in the RIMLF were ensheathed only by fragments of PN, unlike in the control case (Fig 6A–6I). As also seen in other areas, ACAN and HPLN1 staining showed much more fragmented extracellular matrix in the neuropil despite some rather well-preserved PN (Fig 6E).

## Discussion

We describe a patient with profound enduring saccadic palsy following cardiac surgery whose brain showed preserved saccade-generating neurons but disruption of the PN that are thought to be essential for normal functioning of these neurons. These findings raise a number of important issues for discussion regarding the localization of saccade dysfunction, the role of PN, broader damage to the central nervous system, mechanisms of injury, and implications for understanding PN function.

### Damage to the brainstem saccadic network

Brainstem neurons that generate saccades (PBN and OPN) receive descending inputs from cortical eye fields, via the basal ganglia, superior colliculus, and cerebellum [32,33]. The frontal and parietal eye fields lie within arterial watershed areas and are vulnerable to hypoperfusion. Bihemispheric lesions such as strokes affecting cortical eye fields result in ocular motor apraxia, in which all voluntary eye movements–saccades, pursuit, vergence–are impaired, but reflexive movements, such as quick phases of vestibular and optokinetic nystagmus, are preserved [34–37]. Similar voluntary eye movement deficits have been described in children developing chorea following hypothermic cardiopulmonary bypass surgery, attributable to injury of the cerebral cortex and basal ganglia rather than the brainstem [38]. Other saccadic palsy cases, generally with preserved pursuit and vestibular eye movements plus features like dysarthria, suggest brainstem injury despite normal neuroimaging [18,21,22]; one such patient exhibited brainstem and cerebellar hypometabolism as revealed by positron emission tomography [19]. However, most well-studied patients like ours show selective palsy of all voluntary and reflex saccades, with other eye movements preserved [16,23,29].

Additional evidence that this selective saccadic palsy was due to a brainstem process comes from studies of chemical lesions or pharmacological inactivation of the PPRF, RIMLF, or OPN in macaque reporting selective slowing or absence of saccades with spared pursuit and vestibular eye movements [39–42]. Such saccadic slowing is predicted by models of OPN lesions incorporating membrane channels. Miura and Optican [43] proposed that loss of glycine from OPN lesions leads to lower-than-normal EBN activity from reduced T-type calcium channel and NMDA currents, producing slow saccades.

We conclude that our patient’s saccadic palsy was due to dysfunction of the brainstem saccade-generating network of neurons because of her isolated loss of horizontal, vertical, and torsional saccades and reflexive quick phases but preserved pursuit, vergence, and vestibular eye movements. This *selective saccadic palsy* spares ocular motor nuclei and supranuclear pathways from the cerebral hemispheres, basal ganglia, and cerebellum involved in pursuit and vestibular eye movements. While there was some focal loss of cerebellar Purkinje cells, which are susceptible to ischemia or alcohol [44,45], these cells, and the fastigial nucleus to which they project, influence the beginning and ending of saccades to control their size [46] but have only minor effects on saccade speed [47]. The patient’s fastigial nucleus showed strong PN staining as in controls.

### Role of perineuronal nets

The main novel finding of this clinicopathologic study is that neurons in the brainstem reticular formation’s saccadic network appeared histologically normal with preserved synapses despite their loss of function. However, our immunostaining demonstrated that PN surrounding OPN and EBN were absent or severely fragmented. Great methodological care ensured that poor PN staining was not due to effects of preservation, fixation, or postmortem delay by using three antibodies to detect different PN components (even in the same section with immunofluorescence). Furthermore, intact PN were present in adjacent related (IBN, abducens and fastigial nucleus) and unrelated (superior olive) areas. This finding supports the critical role current models place for OPN and EBN in generating normal velocity saccades [33]. It also demonstrates that PN around PBN and OPN play a necessary physiologic role, since their degradation leads to loss of normal function, even while the neurons and their synapses still appear intact based on parvalbumin and synaptophysin immunostaining. It appears that after critical injury, the PN did not regenerate or reorganize, leaving OPN and EBN permanently in their malfunctioning state.

Several functions have been suggested for PN that may underlie the mechanism for malfunction of OPN and EBN [7]. PN could help maintain membrane-channel properties necessary for normal functioning in these highly active neurons. Pharmacological blockade of neuronal activity with tetrodotoxin or various transmitter receptor antagonists resulted in a strongly decreased accumulation of PN in hippocampal cell culture [48]. In this preparation, the removal of PN by chondroitinase ABC does not affect the number and distribution of perisomatic GABAergic contacts in hippocampal cells but does increase excitability of inhibitory interneurons, supporting the importance of PN in regulating interneuronal activity [48]. One mechanism by which PN may affect neuronal function is by serving as rapid local buffers of excess cations in the extracellular space around somatic membranes of fast-spiking neurons [8].

### Broader damage to the central nervous system

Damage to PN might also explain other phenomena following cardiac surgery. PN surround GABAergic projection neurons in portions of the globus pallidus and substantia nigra as well as in subpopulations of striatal and thalamic inhibitory neurons [49]. Hypoxia to these PN could explain the reported childhood cases of choreoathetosis and dyskinesia following hypothermic circulatory arrest with normal neuroimaging [38,50,51]. Epilepsy developed in our patient and others [21,25]. Seizures can be late sequelae of ischemia [52], and hippocampal neurons are particularly vulnerable to ischemia [53]. Hippocampal PN-ensheathed fast-spiking GABAergic interneurons appear to form an electrically-coupled syncytium synchronizing cortical neurons [54]. A post-ischemic rat model demonstrated *in vivo* and *in vitro* spontaneous epileptiform discharges from the CA3 area months after global ischemia, combined with loss of GABAergic interneurons, a finding associated with aberrant sprouting of glutamatergic fibers leading to enhanced synaptic excitation and reduced synaptic inhibition in temporal lobe epilepsy models [55]. Whether selective vulnerability of hippocampal PN-bearing interneurons could cause post-ischemic temporal lobe epilepsy requires additional immunohistochemical studies.

### Mechanism of injury

How could saccade-related PN be damaged during cardiopulmonary bypass while sparing the highly active neurons they enwrap? PN may be particularly vulnerable to hypoxia, as a focal cerebral ischemia rat model found that ischemia damages PN more than the ensheathed neurons in the peri-infarct zone [28]. Unlike the one other pathologic case report that showed ischemic damage to the paramedian pons [16], our patient showed no evidence of infarction. Perhaps our patient’s atherosclerotic basilar artery paramedian perforators were underperfused during circulatory arrest without causing frank infarction but still damaging the brainstem PN. If so, any microglial activation had long since disappeared. Though hypoxic/ischemic injury remains an attractive mechanism given the immediacy of symptoms following cardiopulmonary bypass surgery, other reported cases offer few additional clues to the mechanism of injury to this tiny brainstem region, since such surgeries were generally otherwise uncomplicated, with normal circulatory arrest and total operative time and without hypotension. The fact that this clinical syndrome occurs almost exclusively in patients requiring aortic valve or root surgery for dissection or aneurysm could raise the question of some underlying disorder of the connective tissue matrix that also puts PN at risk. It remains to be determined why OPN and EBN were affected whereas adjacent abducens and IBN, which have similar firing properties to EBN and express glycine like OPN [56], were spared.

### Implications for understanding perineuronal net function

Selective saccadic palsy could be developed into an animal model to clarify the role of PN. Saccades are an ideal system for studying neural control of movement since they are well understood and can be measured precisely [33,57]. The feasibility of experimentally digesting chondroitin sulfate chains of PN with the enzyme chondroitinase ABC [58] has been shown in macaque fastigial nucleus [59]. Similar methods could be applied in the PPRF and RIMLF.

Understanding the role of PN in different disorders may create therapeutic opportunities. Status epilepticus is associated with reduced PN-stabilizing components and aggrecan expression [60]. The role of dysfunctional PN and other extracellular matrix components in epilepsy and other neurological disorders offers a potential therapeutic target [61]. Mutations in extracellular matrix-related genes are associated with several neurological disorders [61] that could be amenable to gene therapy. Pharmacologic manipulation of PN and other extracellular matrix structures to affect neuronal plasticity, stability, or recovery has been discussed for conditions like stroke and Alzheimer disease as well [7,62]. Similarly, targeting brainstem saccade-related regions with therapies to promote PN repair may offer a way to restore function in patients with selective saccadic palsy in which the neurons and synapses are still intact [63].

In summary, this study provides the first evidence that saccadic palsy following cardiac surgery can be caused by damage to supporting tissues surrounding saccadic network neurons rather than to the neurons themselves. It also suggests an accessible model in which eye movements could be used to study the unique role and requirements of PN experimentally. However, some caution is required in interpreting our results, which rest on careful studies of one patient. More studies are required to confirm our findings, including examination of PN in patients without saccadic palsy who have died of exsanguination.

## Supporting Information

S1 Movie Ocular motor examination. To make rapid head-free gaze shifts, the patient used exaggerated head turns associated with blinks; contraversive vestibular slow phases moved the eyes into the corner of the orbits until the head was maximally rotated, and then the eyes slowly drifted toward the target. She made no reflexive saccades. With the head fixed, volitional saccades consisted of extremely slow eye movements that eventually reached the target, except for slightly faster downward saccades. Pursuit was smooth and full in range both horizontally and vertically, even at higher frequencies. Visually enhanced vestibulo-ocular reflex was normal. When she viewed a horizontally rotating optokinetic drum, her eyes became fixed laterally in the orbits without any corrective quick phases. Torsional head rolling produced normal ocular counter-rolling but without any torsional quick phases. See text for additional details not shown in the movie.
(M4V)
